# Supplementary material for: Expansive data, extensive model: Investigating discussion topics around LLM through unsupervised machine learning in academic papers and news
Source: PLoS One. 2024 May 31;19(5):e0304680. doi: 10.1371/journal.pone.0304680 (PMC11142427; doi:10.1371/journal.pone.0304680)
Supplement: Attachment — Submitted filename: Response to Reviewers.docx [file pone.0304680.s001.docx]

**Original Manuscript ID:** PONE-D-24-00612

**Original Article Title: “Expansive Data, Extensive Model: Investigating LLM through Unsupervised Machine Learning in Academic Papers and News**”

**To:** PLOS ONE Access Editor

**Re:** Response to editor and reviewers

Dear Editor,

Thank you for allowing a resubmission of our manuscript, with an opportunity to address the reviewers’ comments.

We are uploading (a) our point-by-point response to the comments (below) (response to reviewers, under “Author’s Response Files*”*), (b) an updated manuscript with track changes (as “Revised Manuscript with Track Changes*”*), and (c) a clean updated manuscript without tracked changes (“Manuscript”*).*

Best regards,

Kim et al.

**Journal requirements # 1:** When submitting your revision, we need you to address these additional requirements. Please ensure that your manuscript meets PLOS ONE's style requirements, including those for file naming. The PLOS ONE style templates can be found at https://journals.plos.org/plosone/s/file?id=wjVg/PLOSOne_formatting_sample_main_body.pdf and https://journals.plos.org/plosone/s/file?id=ba62/PLOSOne_formatting_sample_title_authors_affiliations.pdf.

**Author response:** Thank you for providing guidelines. Based on the PLOS ONE template style you provided, we have reformatted the manuscript to ensure it aligns with PLOS ONE's style requirements. We have also adjusted the file naming accordingly. The details are as follows.

**Author action:** We have made changes to aspects such as indentation and the capitalization of the first letter in subheadings. The main changes we've made are highlighted in red. For more details, please refer to the manuscript itself.

“

**Abstract**

This study presents a comprehensive exploration of topic modeling methods tailored for large language model (LLM) using data obtained from Web of Science and LexisNexis from June 1, 2020, to December 31, 2023. The data collection process involved queries focusing on LLMs, including "Large language model," "LLM," and "ChatGPT." Various topic modeling approaches were evaluated based on performance metrics, including diversity and coherence. latent Dirichlet allocation (LDA), nonnegative matrix factorization (NMF), combined topic models (CTM), and bidirectional encoder representations from Transformers topic (BERTopic) were employed for performance evaluation. Evaluation metrics were computed across platforms, with BERTopic demonstrating superior performance in diversity and coherence across both LexisNexis and Web of Science. The experiment result reveals that news articles maintain a balanced coverage across various topics and mainly focus on efforts to utilize LLM in specialized domains. Conversely, research papers are more concise and concentrated on the technology itself, emphasizing technical aspects. Through the insights gained in this study, it becomes possible to investigate the future path and the challenges that LLMs should tackle. Additionally, they could offer considerable value to enterprises that utilize LLMs to deliver services.

…

**Research on utilization of LLMs in various domain**

…

”

**Journal requirements # 2:** Please note that PLOS ONE has specific guidelines on code sharing for submissions in which author-generated code underpins the findings in the manuscript. In these cases, all author-generated code must be made available without restrictions upon publication of the work. Please review our guidelines at https://journals.plos.org/plosone/s/materials-and-software-sharing#loc-sharing-code and ensure that your code is shared in a way that follows best practice and facilitates reproducibility and reuse.

**Author response:** Thank you for your valuable guidance. We have publicly shared the code and data used in the experiments on GitHub, following the code sharing policy of PLOS ONE. We intend to describe this in the data availability section.

**Author action:** We have uploaded minimal anonymized data and code at public repository. Additionally, we intend to describe this in the data availability.

**Journal requirements # 3:** Thank you for stating the following financial disclosure: [This study was supported by a National Research Foundation of Korea (NRF) (http:// nrf.re.kr/eng/index) grant funded by the Korean government (RS-2023-00208278).]. Please state what role the funders took in the study. If the funders had no role, please state: ""The funders had no role in study design, data collection and analysis, decision to publish, or preparation of the manuscript."" If this statement is not correct you must amend it as needed. Please include this amended Role of Funder statement in your cover letter; we will change the online submission form on your behalf.

**Author response:** Thank you for providing guidelines. Based on the guidance, we have included the amended role of funder statement in the cover letter regarding the funder's role. The details are as follows.

**Author action:** We have made modifications to the parts highlighted in red in cover letter below.

“

…

**Role of Funder**

The funders had no role in study design, data collection and analysis, decision to publish, or preparation of the manuscript.

”

**Journal requirements # 4:** We note that you have indicated that there are restrictions to data sharing for this study. PLOS only allows data to be available upon request if there are legal or ethical restrictions on sharing data publicly. For more information on unacceptable data access restrictions, please see http://journals.plos.org/plosone/s/data-availability#loc-unacceptable-data-access-restrictions.

Before we proceed with your manuscript, please address the following prompts:

a) If there are ethical or legal restrictions on sharing a de-identified data set, please explain them in detail (e.g., data contain potentially identifying or sensitive patient information, data are owned by a third-party organization, etc.) and who has imposed them (e.g., a Research Ethics Committee or Institutional Review Board, etc.). Please also provide contact information for a data access committee, ethics committee, or other institutional body to which data requests may be sent.

b) If there are no restrictions, please upload the minimal anonymized data set necessary to replicate your study findings to a stable, public repository and provide us with the relevant URLs, DOIs, or accession numbers. For a list of recommended repositories, please see
https://journals.plos.org/plosone/s/recommended-repositories. You also have the option of uploading the data as Supporting Information files, but we would recommend depositing data directly to a data repository if possible.

We will update your Data Availability statement on your behalf to reflect the information you provide.

**Author response:** Thank you for your valuable guidance. We have publicly shared the code and data used in the experiments on GitHub, following the code sharing policy of PLOS ONE. We intend to describe this in the data availability section.

**Author action:** We have uploaded minimal anonymized data and code at public repository. Additionally, we intend to describe this in the data availability.

**Journal requirements # 5:** In the online submission form, you indicated that [The datasets utilized or examined in this study can be obtained from the corresponding author upon a reasonable request.]. All PLOS journals now require all data underlying the findings described in their manuscript to be freely available to other researchers, either 1. In a public repository, 2. Within the manuscript itself, or 3. Uploaded as supplementary information. This policy applies to all data except where public deposition would breach compliance with the protocol approved by your research ethics board. If your data cannot be made publicly available for ethical or legal reasons (e.g., public availability would compromise patient privacy), please explain your reasons on resubmission and your exemption request will be escalated for approval.

**Author response:** Thank you for your valuable guidance. We have publicly shared the code and data used in the experiments on GitHub, following the code sharing policy of PLOS ONE. We intend to describe this in the data availability section.

**Author action:** We have uploaded minimal anonymized data and code at public repository. Additionally, we intend to describe this in the data availability.

**Reviewer#1, Concern # 1:** The research article offers an extensive investigation into topic modeling techniques customized for large language models (LLMs), employing data sourced from LexisNexis and Web of Science. The authors are requested to address the following queries: 1) The manuscript is effectively presented with a strong technical foundation. The methodology description and result discussion sections are compelling and appropriate. 2) The authors should address grammatical errors scattered throughout the manuscript, as well as improve the alignment of equations and enhance the quality of figures to uphold the standards and integrity of the journal.

**Author response:** Thank you for your valuable comments. Following your suggestions, we have worked on reducing grammatical errors, modifying the numbering and format of the equations and to fit in the PLOS ONE manuscript guidelines. Additionally, efforts were made to enhance the visibility of the figures and the detailed explanations. Only the main points have been included in the letter. Please refer to the manuscript for detailed information. The details are as follows.

**Author action:** We have made modifications to the parts highlighted in red in the manuscript below.

“

…

**Topic analysis based on performance evaluation metrics**

…

**Evaluation metrics**

…

$Topic Diversity=\frac{M}{N\times k}$	(1)

$N{PMI\left( w_{i}, w_{j} \right)}^{\gamma}=\left( \frac{PMI\left( w_{i}, w_{j} \right)}{-\log P(w_{i}, w_{j})+\epsilon} \right)^{\gamma}$	(2)

$PMI\left( w_{i}, w_{j} \right)=log\frac{P\left( w_{i}, w_{j} \right)+\epsilon}{P(w_{i})\cdot P(w_{j})}$	(3)

”

**Reviewer#1, Concern # 2:** It is advisable for the authors to emphasize the complexity of the proposed method to assess both its robustness and efficiency.

**Author response:** Thank you for your valuable comments. Following your suggestion, paragraphs describing each baseline model and BERTopic were added to illustrate the complexity of the proposed methodology and ensure the validity and the efficiency of the approach. Additionally, please note that Fig 2 comparing the models has been added in the Experiment section. The details are as follows.

**Author action:** We have made modifications to the parts highlighted in red in the manuscript below.

“

…

**Topic modeling models**

…

The first topic modeling algorithm employed is LDA. LDA is a statistical model for topic modeling that assumes a combination of topics for a document collection and models how each document is distributed across these topics [28]. The LDA is widely used to derive topics from text data [29]. The time complexity of the LDA based on Gibbs sampling and can be expressed as follows [30].

$Time complexity \left( LDA \right)=O(N\cdot M\cdot K\cdot iter\#)$	(4)

In the equation, $N$ and $M$ refer to the number of documents and words, $K$ is the number of topics, and $iter\#$ is the number of repetitions.

The second topic modeling method utilized is NMF. NMF involves representing a nonnegative matrix as the result of multiplying two nonnegative matrices of lower rank [31]. When applied to term frequency-inverse document frequency transformed data, NMF breaks down the matrix into two smaller matrices. Due to its ability to provide semantically significant results in clustering tasks, NMF has become popular as both a segmentation and topic modeling technique, particularly in document analysis [32]. The time complexity of NMF is as follows.

$Time complexity \left( NMF \right)=O(n^{2}\cdot m\cdot k)$	(5)

In the equation, $n^{2}$ is the number of documents corresponding to the number of rows of the data matrix, $m$ is the number of words corresponding to the column, and $k$ is the number of topics [33].

The third methodology applied is combined topic model (CTM). CTM refers to composite topic modeling approaches that combine the bag of words with sentence BERT (SBERT), a document representation technique from pre-trained language models [34]. CTM aims to derive topics from documents by employing the contextual expression of BERT’s document embeddings and merging them with the unsupervised learning abilities inherent in conventional topic models. Since the time complexity of CTM depends on the BERT model, it depends on the input token $O(n)$. However, since the CTM incorporates the LDA with products of experts (ProdLDA) model with BERT, the time complexity stemming from Gibbs sampling is considered together.

The last algorithm utilized is BERTopic. BERTopic utilizes BERT embeddings alongside c-TF-IDF to create compact clusters that preserve essential words within topics [35]. Furthermore, BERTopic's modular structure facilitates the integration and utilization of various algorithms. By employing contextual word and sentence vector representations, BERTopic leverages semantic properties to position similar texts closely in the vector space. It leverages SBERT for document embedding, which generates dense vectors for sentences and paragraphs. Dimensionality reduction is then executed using uniform manifold approximation and projection (UMAP), followed by cluster creation via hierarchical density-based spatial clustering of applications with noise (HDBSCAN) [18]. Prior to computing c-TF-IDF, the authors first utilized CountVectorizer to generate topic representation. This preprocessing step is intended to reduce the dimensions of the c-TF-IDF matrix. Subsequently, topic representation is obtained from each cluster using c-TF-IDF. In this approach, each clustered document is treated as a single document, with inverse document frequency substituted by inverse class frequency to assess the relevance of words for the class. This methodology reinforces the importance of words within a cluster, leading to the creation of a topic-word distribution for each document cluster. This procedure simplifies the process of identifying representative words for the respective topics. The time complexity of BERTopic is $O(n)$, reflecting the complexity of BERT.

…

**Evaluation metric comparison for platform-specific topic modeling models**

…

**Fig 2. Detailed schematic diagram of the comparison of evaluation metrics between models**

”

***Newly added references***

28.	Blei D. M, Ng A. Y, Jordan M. I. Latent dirichlet allocation. Journal of machine Learning research. 2003; 3: 993-1022.

30.	Shao D, Li C, Huang C, Xiang Y, Yu Z. A news classification applied with new text representation based on the improved LDA. Multimedia tools and applications. 2022; 81 (15): 21521-21545.

31.	Lee D. D, Seung H. S. Learning the parts of objects by non-negative matrix factorization. nature. 1999; 401: 788-791.

32.	Kuang D, Choo J, Park H. Nonnegative matrix factorization for interactive topic modeling and document clustering. Partitional clustering algorithms. 2015; doi: 10.1007/978-3-319-09259-1_7.

33.	Lopes N, Ribeiro B, Lopes N, Ribeiro B. Non-negative matrix factorization (NMF). Machine Learning for Adaptive Many-Core Machines-A Practical Approach. 2015; doi: 10.1007/978-3-319-06938-8_7.

**Reviewer#1, Concern # 3:** When using LLMs for topic modeling, it's essential to consider the computational resources required for processing large volumes of text data and the interpretability of the topics extracted.

**Author response:** Thank you for your valuable statements. Our research focuses not on utilizing LLMs for topic modeling, but on conducting topic modeling for the query "LLM." Nonetheless, we have included discussions on time complexity and the advantages of utilizing BERTopic. To consider the computational resources of the models, we added the time complexity for each model in the "Topic modeling models" section. Comparing the described time complexities, models involving Gibbs sampling such as LDA and CTM require significant resources, while NMF based on matrix factorization and BERTopic, which depends on the number of input data, perform relatively fewer operations. Additionally, NMF, which approximates a given matrix into two low-rank matrices using the document-word matrix, faces difficulty in explicitly representing topics, resulting in low interpretability. On the other hand, the BERTopic model, which considers context during learning, offers high interpretability and clear clustering of topics. Therefore, it is justified for us to use BERTopic to analyze large-scale domain-specific text data. The details are as follows.

**Author action:** We have made modifications to the parts highlighted in red in the manuscript below.

“

…

**Topic modeling models**

The first topic modeling algorithm employed is LDA. LDA is a statistical model for topic modeling that assumes a combination of topics for a document collection and models how each document is distributed across these topics [28]. The LDA is widely used to derive topics from text data [29]. The time complexity of the LDA based on Gibbs sampling and can be expressed as follows [30].

$Time complexity \left( LDA \right)=O(N\cdot M\cdot K\cdot iter\#)$	(4)

In the equation, $N$ and $M$ refer to the number of documents and words, $K$ is the number of topics, and $iter\#$ is the number of repetitions.

The second topic modeling method utilized is NMF. NMF involves representing a nonnegative matrix as the result of multiplying two nonnegative matrices of lower rank [31]. When applied to term frequency-inverse document frequency transformed data, NMF breaks down the matrix into two smaller matrices. Due to its ability to provide semantically significant results in clustering tasks, NMF has become popular as both a segmentation and topic modeling technique, particularly in document analysis [32]. The time complexity of NMF is as follows.

$Time complexity \left( NMF \right)=O(n^{2}\cdot m\cdot k)$	(5)

In the equation, $n^{2}$ is the number of documents corresponding to the number of rows of the data matrix, $m$ is the number of words corresponding to the column, and $k$ is the number of topics [33].

The third methodology applied is combined topic model (CTM). CTM refers to composite topic modeling approaches that combine the bag of words with sentence BERT (SBERT), a document representation technique from pre-trained language models [34]. CTM aims to derive topics from documents by employing the contextual expression of BERT’s document embeddings and merging them with the unsupervised learning abilities inherent in conventional topic models. Since the time complexity of CTM depends on the BERT model, it depends on the input token $O(n)$. However, since the CTM incorporates the LDA with products of experts (ProdLDA) model with BERT, the time complexity stemming from Gibbs sampling is considered together.

The last algorithm utilized is BERTopic. BERTopic utilizes BERT embeddings alongside c-TF-IDF to create compact clusters that preserve essential words within topics [35]. Furthermore, BERTopic's modular structure facilitates the integration and utilization of various algorithms. By employing contextual word and sentence vector representations, BERTopic leverages semantic properties to position similar texts closely in the vector space. It leverages SBERT for document embedding, which generates dense vectors for sentences and paragraphs. Dimensionality reduction is then executed using uniform manifold approximation and projection (UMAP), followed by cluster creation via hierarchical density-based spatial clustering of applications with noise (HDBSCAN) [18]. Prior to computing c-TF-IDF, the authors first utilized CountVectorizer to generate topic representation. This preprocessing step is intended to reduce the dimensions of the c-TF-IDF matrix. Subsequently, topic representation is obtained from each cluster using c-TF-IDF. In this approach, each clustered document is treated as a single document, with inverse document frequency substituted by inverse class frequency to assess the relevance of words for the class. This methodology reinforces the importance of words within a cluster, leading to the creation of a topic-word distribution for each document cluster. This procedure simplifies the process of identifying representative words for the respective topics. The time complexity of BERTopic is $O(n)$, reflecting the complexity of BERT.

”

***Newly added references***

28.	Blei D. M, Ng A. Y, Jordan M. I. Latent dirichlet allocation. Journal of machine Learning research. 2003; 3: 993-1022.

30.	Shao D, Li C, Huang C, Xiang Y, Yu Z. A news classification applied with new text representation based on the improved LDA. Multimedia tools and applications. 2022; 81 (15): 21521-21545.

31.	Lee D. D, Seung H. S. Learning the parts of objects by non-negative matrix factorization. nature. 1999; 401: 788-791.

32.	Kuang D, Choo J, Park H. Nonnegative matrix factorization for interactive topic modeling and document clustering. Partitional clustering algorithms. 2015; doi: 10.1007/978-3-319-09259-1_7.

33.	Lopes N, Ribeiro B, Lopes N, Ribeiro B. Non-negative matrix factorization (NMF). Machine Learning for Adaptive Many-Core Machines-A Practical Approach. 2015; doi: 10.1007/978-3-319-06938-8_7.

**Reviewer#1, Concern # 4:** Additionally, fine-tuning LLMs on domain-specific text corpora may improve the quality of topic modeling results for specialized domains. The authors should address this.

**Author response:** Thank you for your valuable statements. Our research is oriented towards conducting topic modeling for the query "LLM," rather than utilizing LLMs for topic modeling. Nevertheless, we have validated the appropriateness of fine-tuning Modified BERTopic for the text collected using the "LLM" query. Specifically, we implemented a 'modified BERTopic' to analyze data specific to the LLM domain. This is the same as the fine-tuning process. In detail, a revised BERTopic diagram and contents containing these details are attached. This is further explained in the Topic Modeling Models section. The details are as follows.

**Author action:** We have made modifications to the parts highlighted in red in the manuscript below.

“

…

**Topic modeling models**

…

The last algorithm utilized is BERTopic. BERTopic utilizes BERT embeddings alongside c-TF-IDF to create compact clusters that preserve essential words within topics [35]. Furthermore, BERTopic's modular structure facilitates the integration and utilization of various algorithms. By employing contextual word and sentence vector representations, BERTopic leverages semantic properties to position similar texts closely in the vector space. It leverages SBERT for document embedding, which generates dense vectors for sentences and paragraphs. Dimensionality reduction is then executed using uniform manifold approximation and projection (UMAP), followed by cluster creation via hierarchical density-based spatial clustering of applications with noise (HDBSCAN) [18]. Prior to computing c-TF-IDF, the authors first utilized CountVectorizer to generate topic representation. This preprocessing step is intended to reduce the dimensions of the c-TF-IDF matrix. Subsequently, topic representation is obtained from each cluster using c-TF-IDF. In this approach, each clustered document is treated as a single document, with inverse document frequency substituted by inverse class frequency to assess the relevance of words for the class. This methodology reinforces the importance of words within a cluster, leading to the creation of a topic-word distribution for each document cluster. This procedure simplifies the process of identifying representative words for the respective topics. The time complexity of BERTopic is $O(n)$, reflecting the complexity of BERT.

”

**Reviewer#1, Concern # 5:** Additionally, the authors are suggested to discuss graph-based or deep learning topic modeling techniques to underscore the significance of these approaches.

**Author response:** Thank you for your valuable statements. This study utilized BERTopic for analysis, and conducted comparisons with two traditionally used models (LDA, NMF) and a deep learning-based approach (CTM) to demonstrate the superiority of the model. The comparative experiments were performed using Coherence and Diversity metrics, where BERTopic performed exceptionally well. Due to its utilization of pre-trained language models like SBERT, BERTopic is categorized within the domain of deep learning methodologies. Furthermore, although it was possible to perform graph-based topic modeling, we did not use that methodology. The reasons are as follows: Firstly, BERTopic generates word and sentence embeddings considering the context, thereby grasping the semantic relationships among words. On the other hand, graph-based learning overlooks the context comprehensively by focusing on relationships between neighboring nodes. Secondly, BERTopic employs clustering algorithms to group similar sentences, whereas graph-based models analyze relationships between nodes, which may not align with the research intent of summarizing topics through clustering. Thirdly, BERTopic extracts important keywords for each topic, enhancing interpretability and facilitating better understanding and summarization of the entire document. For these reasons, we interpreted the data based on BERTopic.

**Author action:** We have responded to the reviewer's comments sincerely and provided detailed explanations with supporting reason.

**Reviewer#1, Concern # 6:** The authors are advised to incorporate state-of-the-art papers in the experimental section for comparative analysis, thus elucidating the significance of their proposed models.

**Author response:** Thank you for your valuable comments. We would like to explain the rationale and validity behind our selection of the baseline. We have compared BERTopic with both traditional and relatively new models, and utilized its superiority following the validation process. Specifically, we employed LDA and NMF as baselines due to their wide usage in the field of topic modeling. Additionally, CTM, being one of the newer models and a deep learning-based one, was included in the comparison to verify the suitability of utilizing BERTopic. Secondly, the reason why BERTopic stands out as the optimal choice lies in its flexibility and performance. BERTopic generates context-aware word and sentence embeddings based on the latest deep learning technologies such as SBERT. This allows for a better understanding of semantic similarity between words, and forms topics by clustering similar sentences. These detailed functions have a wide range of applicability because they vary depending on the specific domain, and can be optimized to suit the domain. In fact, other models besides BERTopic have been proposed to date, but BERTopic is the best in terms of applicability. Finally, we added future plans for verification of new SOTA models in the limitations section of the conclusion. If a better and more versatile model than BERTopic is proposed, we will perform additional experiments using those models and compare the results to verify the performance of our model more objectively. The details are as follows.

**Author action:** We have made modifications to the parts highlighted in red in the manuscript below.

“

…

**Topic modeling models**

…

The third methodology applied is combined topic model (CTM). CTM refers to composite topic modeling approaches that combine the bag of words with sentence BERT (SBERT), a document representation technique from pre-trained language models [34]. CTM aims to derive topics from documents by employing the contextual expression of BERT’s document embeddings and merging them with the unsupervised learning abilities inherent in conventional topic models. Since the time complexity of CTM depends on the BERT model, it depends on the input token $O(n)$. However, since the CTM incorporates the LDA with products of experts (ProdLDA) model with BERT, the time complexity stemming from Gibbs sampling is considered together.

The last algorithm utilized is BERTopic. BERTopic utilizes BERT embeddings alongside c-TF-IDF to create compact clusters that preserve essential words within topics [35]. Furthermore, BERTopic's modular structure facilitates the integration and utilization of various algorithms. By employing contextual word and sentence vector representations, BERTopic leverages semantic properties to position similar texts closely in the vector space. It leverages SBERT for document embedding, which generates dense vectors for sentences and paragraphs. Dimensionality reduction is then executed using uniform manifold approximation and projection (UMAP), followed by cluster creation via hierarchical density-based spatial clustering of applications with noise (HDBSCAN) [18]. Prior to computing c-TF-IDF, the authors first utilized CountVectorizer to generate topic representation. This preprocessing step is intended to reduce the dimensions of the c-TF-IDF matrix. Subsequently, topic representation is obtained from each cluster using c-TF-IDF. In this approach, each clustered document is treated as a single document, with inverse document frequency substituted by inverse class frequency to assess the relevance of words for the class. This methodology reinforces the importance of words within a cluster, leading to the creation of a topic-word distribution for each document cluster. This procedure simplifies the process of identifying representative words for the respective topics. The time complexity of BERTopic is $O(n)$, reflecting the complexity of BERT.

…

**Conclusion**

…

However, this study carries several limitations for future exploration. This research exclusively processed English-language data, while countries exhibit diverse LLM related perceptions and text. Furthermore, subsequent research could investigate public interest from social media text data, which was not covered in this study. In addition, further comparative analysis with emerging deep learning-based topic modeling methods should be supplemented in future studies.

”

**Reviewer#2, Concern # 1:** I suggest authors to reconsider the title of this research. If I understood this correctly, this research is about comparing different topic modelling techniques. After reading this particle, I am still having a difficulty in finding on which part of this paper actually deals with "investigating LLM through unsupervised machine learning..."

**Author response:** We appreciate your valuable comments. Based on your suggestion, we have made an adjustment to the title to better match the content of the research. The details are as follows.

**Author action:** We have made modifications to the parts highlighted in red in the manuscript below.

“

Expansive data, extensive model: Investigating discussion topics around LLM through unsupervised machine learning in academic papers and news

”

**Reviewer#2, Concern # 2:** The motivation and novelty of this research remain somewhat unclear in the manuscript. The authors state that the goal of this research is to enhance the recognition and derivation of insights from large language models (LLMs) across three distinct platforms. But this research goal is too broad and general. Instead, authors need to specify their aim of research and provide full description on why this research is needed. In addition, it is essential that the Introduction section specifies and clarifies which three unique platforms are under investigation.

**Author response:** We appreciate your valuable comments. Following your suggestion, we have developed on the research objectives and significance in the last part of the introduction. In detail, the aim of this study is to use machine learning to identify various topics related to LLMs discussed in both media and academia, and to uncover the underlying meanings. The importance of such analysis lies in its value as feedback within industries that utilize LLMs and in its ability to discover new insights. Additionally, it can be utilized when adjusting product or service strategies. The details are as follows.

**Author action:** We have made modifications to the parts highlighted in red in the manuscript below.

“

…

**Introduction**

…

This research aims to enrich the body of knowledge by employing data mining with unsupervised machine learning methods to recognize and derive insights from LLM-related text within two unique platforms: LexisNexis representing the media and Web of Science representing academia, contributing to perception and insight into the field. Ultimately, this study discovers the unique meanings inherent within LLM and enhance both commercial and academic value. The significance of such analysis lies in its ability to provide value as feedback within industries relying on LLMs, thereby facilitating the exploration of new insights. Additionally, the insight from the experiment can be utilized when adjusting relevant product or service strategies.

”

**Reviewer#2, Concern # 3:** In the Related Works section, the authors present a series of studies on the application of large language models (LLMs) across various domains, and another series of studies on Topic Modeling. This former content might be more appropriate for the Introduction.

**Author response:** We appreciate your valuable comments. We would like to explain why we believed these aspects are more suitable for the replated works section first, and then we will address the changes that have been made in the manuscript.

The “2.1 Research on utilization of LLMs in various domains” provides insight into the application of LLM across various domains, enabling authors to identify overall trends in LLM and acquire information about ongoing research. Analyzing the “2.2 topic modeling” part is necessary as it is comparable to reviewing the historical aspect of our core methodology in this study. In particular, including the approach of using topic modeling for analysis in the Related Works section confirms the justification for choosing the research methodology and illustrates the benefits of utilizing this method in the study. To provide more detailed information, we have added content to the concluding sections of each related works. The details are as follows.

**Author action:** We have made modifications to the parts highlighted in red in the manuscript below.

“

…

**Related works**

**Research on utilization of LLMs in various domain**

…

In summary, these studies have underscored the adaptability of LLMs, affirming their ability to be widely utilized in diverse field and demonstrating the potential benefits of employing LLMs across diverse domains such as problem-solving, automation, and natural language comprehension.

**Topic modeling**

…

[13] performed a comparative study of different topic models, encompassing latent Dirichlet algorithm (LDA), correlated topic model, hierarchical Dirichlet process (HDP), and Dirichlet multinomial regression (DMR). The authors employed adolescent drug use and depression as queries for their analysis. [14] employed LDA, nonnegative matrix factorization (NMF), and bidirectional encoder representations from Transformers topic (BERTopic) on Twitter posts, performing a comparative assessment for each individual topic modeling algorithm. [15] harnessed BERTopic embeddings and class-based term frequency inverse document frequency (c-TF-IDF) to created dense clusters, conducting an analysis of the top themes associated with disparities in cancer care and the model was evaluated using topic coherence scores. [16] experimented with comparing the coherence values using LDA and NMF on literature related to coronavirus disease (COVID-19) to determine which topic model produces a higher-quality representation. [17] utilized LDA analysis on Twitter data to assess the efficiency of the energy market. Subsequently, the derived topic was employed in a classification model to evaluate the accuracy of predicting market movements. [18] used BERTopic on environmental, social, and governance text data to analyze comprehensive evaluation. [19] employed BERTopic to analyze text data on Bitcoin from LexisNexis, Web of Science, and Reddit, representing the media, academia, and public. Additionally, dynamic topic modeling (DTM) was used to monitor changing themes overtime. As illustrated, topic modeling has aided in extracting clusters and meanings from large text datasets. Consequently, researchers across various fields were able to comprehend and analyze the main themes and insights within large document collections, thus demonstrating the applicability of topic modeling across diverse domains.

”

**Reviewer#2, Concern # 4:** Data Collection section needs to be rewritten with better description and explanation. In the manuscript, authors introduced list of search words, but this is not sufficient. How did authors collect data? Did they use search query for all title, abstract, keyword, etc.? What are the restrictions of data search? any geographical restriction? what + Which LexisNexis data source did authors use? Since LexisNexis is well known with its patent database, authors need to clarify this.

**Author response:** We appreciate your valuable comments. Following your advice, we have added additional explanations to the data collection section. Specifically, we have clarified the data collection methods, the range of queries applied, the format of collected data, the necessary conditions for collection, and the platforms used for collection. The details are as follows.

**Author action:** We have made modifications to the parts highlighted in red in the manuscript below.

“

…

**Data collection**

To collect as diverse data as possible, data was gathered through queries related to LLM and the name of prominent LLM technology. Specifically, the goal was to search and collect all the searchable fields containing the query "Large language model," "LLM," and "ChatGPT" as search terms. While there are numerous LLMs beyond ChatGPT, to reduce noise and derive significant results, only the most representative model name was utilized.

Gathering data from LexisNexis and Web of Science, the authors aimed to understand the perceptions toward LLM across news and academic. In detail, the LexisNexis data was collected by accessing Lexis+ and subsequently using a Python scraper to gather .DOCX format files, which were then preprocessed into comma-separated values format. For the Web of Science data, downloads were performed for .XLSX files. The access to LexisNexis database data may require an institutional paid subscription. Specifically, LexisNexis provided complete news articles from major news publications over the world and Web of Science contributed academic paper abstracts.

”

**Reviewer#2, Concern # 5:** One of my concerns regarding this research is the comparison of topic modelling techniques. LDA and CTM are widely known frequency-based topic modelling technique and their pros and cons are widely known. Unlike them, BERTopic is a topic modelling technique relies on text embedding vector with clustering and data dimension techniques. As the result of this research shows, it is somewhat obvious that BERTopic outperforms compared to other two. In addition, the way BERTopic decides number of topic is totally different from how other two does. Due to this fact, I would suggest authors to compare either frequency-based approaches (such as LDA, DTM, CTM, etc.) or embedding-based approaches.

**Author response:** We appreciate your valuable comments. Firstly, we would like to explain why we used LDA and CTM as baselines, and then inform you that we conducted comparative experiments by adding NMF.

Firstly, LDA is the most widely used topic modeling technique and the most traditional method. This is considered a three-level hierarchical Bayesian model. NMF is a linear algebraic algorithm that uses matrix factorization. Using the term-document matrix as input, the topic is extracted by decomposing it into a term-topic matrix and a topic-document matrix. LDA and NMF modeling techniques perform dimensionality reduction to process complex text, and the BERTopic method we used in this study also includes a dimensionality reduction method called UMAP [14]. Therefore, for the superiority of using BERTopic, it is essential to compare it with similar but traditional techniques such as LDA and NMF. Furthermore, CTM refers to a combined topic modeling approach that combines bag of words with sentence BERT (SBERT). Since CTM also utilizes BERT-based embeddings, it can be considered an embedding-based approach. Therefore, we have included CTM as part of the baseline. Furthermore, since coherence and diversity are metrics used to evaluate the performance of topic modeling regardless of the method employed, we believe it is a valid approach to compare different methods using these metrics [14]. The details are as follows.

**Author action:** We have described the reason for choosing these models as baselines in response to the points you raised. Additionally, we have made modifications to the parts highlighted in red in the manuscript below.

“

…

**Evaluation metrics**

…

The second evaluation metric is topic coherence value. The coherence value examines how well words within a topic relate in meaning [25]. Therefore, achieving a higher coherence value is desirable for connecting similar words within each topic group. Among diverse coherence measures, the authors selected for $c\_v$ based on the advantage presented in [26,27]. $c\_v$ uses a sliding window of size 110 to calculate the co-occurrence count of the given words. This count is used to compute the normalized pointwise mutual information (NPMI) between all keywords, resulting in a set of vectors for each key word. The one-set segmentation of primary queries calculate the similarity between each key query vector the aggregate of all keyword vectors. Consequently, cosine similarity is used for this similarity measurement and Coherence is the average of these similarities. The equation for NPMI is expressed as follows:

$N{PMI\left( w_{i}, w_{j} \right)}^{\gamma}=\left( \frac{PMI\left( w_{i}, w_{j} \right)}{-\log P(w_{i}, w_{j})+\epsilon} \right)^{\gamma}$	(2)

$PMI\left( w_{i}, w_{j} \right)=log\frac{P\left( w_{i}, w_{j} \right)+\epsilon}{P(w_{i})\cdot P(w_{j})}$	(3)

The probability is calculated through the consideration of the frequency of word co-occurrences, denoted as $w_{i}$ and $w_{j}$. $\epsilon$ represents a smoothing count value implemented to avoid the numerator from reaching zero and $\gamma$ determines the weight of NPMI.

…

**Evaluation metric comparison for platform-specific topic modeling models**

…

**Fig 2. Comparison of coherence values based on the number of topics from the LexisNexis data**

**Fig 3. Comparison of coherence values based on the number of topics from the Web of Science data**

”

***Newly added references***

26.	Röder M, Both A, Hinneburg A. Exploring the space of topic coherence measures. In Proceedings of the eighth ACM international conference on Web search and data mining. 2015; doi: 10.1145/2684822.2685324.

27.	Syed S, Spruit M. Full-text or abstract? examining topic coherence scores using latent dirichlet allocation. In 2017 IEEE International conference on data science and advanced analytics (DSAA). 2017; doi: 10.1109/DSAA.2017.61.

**Reviewer#2, Concern # 6:** Authors need to make a significant improvement in conclusion. Rather than just summarizing the research, authors need to mention how this research contribute to the main stream and its unique value.

**Author response:** We appreciate your valuable comments. Based on your advice, we have emphasized the contributions and value of this study in the conclusion section. Specifically, we have described the characteristics of both media and academia, highlighting their differences. Additionally, we have outlined the strengths of utilizing both datasets. Subsequently, we have elaborated on the value that this research can provide and emphasized the value. We have also added references to support these points. The details are as follows.

**Author action:** We have made modifications to the parts highlighted in red in the manuscript below.

“

…

**Conclusion**

Despite the swift expansion and extensive use of LLMs across various fields, there remains a significant gap in research concerning topic modeling conducted specifically for LLMs. Consequently, examining and comprehending text associated with LLMs, while investigating insights and perceptions, represents an essential step towards promoting the progress and growth of LLMs.

This study gathered unstructured text data from LexisNexis and Web of Science, representing media and academia, respectively. The data used in the experiment spans from June 2020 when GPT-3 was first released to January 2024. Additionally, the authors first compared the evaluation metrics of four topic modeling methodologies to identify and utilize the optimal methodology for each target platform.

These results have the following theoretical implications. First, media outlets such as news increase public awareness by emphasizing the application of LLM. This promotes public understanding and interest in the professional field [66]. Second, an emphasis on technical aspects in academia provide valuable information to experts in the field and play an important role in technological advancement. Third, the balance between diversity and technical depth between news articles and research papers maintains harmony between expertise and public consciousness, which contributes to effective communication and knowledge transfer [25].

The practical implications can be summarized as follows. This study has important value for companies that provide services by applying LLM. For instance, companies serving the general public should ensure a range of diverse services. Conversely, enterprises integrating LLM with specialized fields like academia should prioritize offering sophisticated and advanced technologies.

Consequently, the findings from the experiment demonstrate that news articles provide even coverage of diverse topics, emphasizing the application of LLM in specialized fields. In contrast, research papers are more compact, concentrating primarily on the technology itself and emphasizing technical aspects.

However, this study carries several limitations for future exploration. This research exclusively processed English-language data, while countries exhibit diverse LLM related perceptions and text. Furthermore, subsequent research could investigate public interest from social media text data, which was not covered in this study. In addition, further comparative analysis with emerging deep learning-based topic modeling methods should be supplemented in future studies.

”

***Newly added references***

66.	Van der Wurff R. Do audiences receive diverse ideas from news media? Exposure to a variety of news media and personal characteristics as determinants of diversity as received. European Journal of Communication. 2011; 26 (4): 328-342.

**Reviewer#3, Concern # 1:** The paper has a little bit misleading title. I expected from the title, that there will be an overview of LLM technologies themselves, and not an overview of discussion areas of LLM as such. Anyway, the paper may be of interest for a number of readers. I would still recommend extending the title by adding a word: Investigating LLM -> Investigating Discussion Topics Around LLM … or something like this.

**Author response:** We appreciate your valuable comments. Following your suggestion, we have revised the title to better align with the content of the research. The details are as follows.

**Author action:** We have made modifications to the parts highlighted in red in the manuscript below.

“

Expansive data, extensive model: Investigating discussion topics around LLM through unsupervised machine learning in academic papers and news

”

**Reviewer#3, Concern # 2:** A drawback of the manuscript is that it is not self-contained. The evaluation metric “diversity” is not defined in the paper. But this metric is crucial when claiming BERT superiority.

**Author response:** We appreciate your valuable comments. According to the comments you provided, we have added detailed definition, explanations, equation, and information regarding topic diversity, as well as references to support it. The details are as follows.

**Author action:** We have made modifications to the parts highlighted in red in the manuscript below.

“

…

**Evaluation metrics**

The authors employed two evaluation metrics for comparing topic modeling method’s performance.

The first evaluation metric is topic diversity. The diversity evaluates the range of topics included, referring in topic modeling to the extent to which each topic represents different subjects or concepts. Topic diversity ranges from 0 to 1 and is one of the important factors in evaluating the quality of a topic model [23]. According to [24], an excessively large number may lead to topics that share commonalities, with overlapping key queries. Conversely, an excessively small number may lead to complex and expansive topics that pose challenges in interpretation. Therefore, if the diversity falls within the range of 0.7 to 0.9, it can be considered an appropriate diversity value.

Topic equation for topic diversity can be stated as follows:

$Topic Diversity=\frac{M}{N\times k}$	(1)

Where $M$ represents the aggregate count of unique words extracted across all topics. $N$ denotes the total number of topics in the model output, and $k$ signifies the count of top words extracted from each individual topic.

”

***Newly added references***

24.	Abdelrazek A, Eid Y, Gawish E, Medhat W, Hassan A. Topic modeling algorithms and applications: A survey. Information Systems. 2023; 112 (102131).

**Reviewer#3, Concern # 3:** The choice of the number of clusters/topics is not explained. I guess that it is based on coherence, but BERT does not have the best coherence at 7 clusters for both datasets. Then why not to choose different number of clusters? In particular, what does it mean that “The crucial point to note is that anomaly occurred when the number of topics was set to three in LexisNexis data.”

**Author response:** We appreciate your valuable comments. Having high coherence indicates that there is a high consistency of words within documents belonging to a single topic. However, when three topics are chosen in topic modeling, it tends to favor larger clusters, limiting the ability to derive a diverse range of topics and resulting in abstract, large-sized clusters. Consequently, conducting detailed analysis on the collected text data becomes impractical. The term "anomaly" was originally used to describe these aspects. However, after reviewing the reviewers' comments, we have added additional model to demonstrate the validity and efficiency of our model selection, and subsequently conducted the additional experiment. Furthermore, to extract better results while reviewing existing literature, we replaced the previously used evaluation metric, c_uci, with c_v and conducted the assessment again, accordingly extracting coherence values. We have also added references supporting these changes. The details are as follows.

**Author action:** We have made modifications to the parts highlighted in red in the manuscript below.

“

…

**Evaluation metrics**

…

The second evaluation metric is topic coherence value. The coherence value examines how well words within a topic relate in meaning [25]. Therefore, achieving a higher coherence value is desirable for connecting similar words within each topic group. Among diverse coherence measures, the authors selected for $c\_v$ based on the advantage presented in [26,27]. $c\_v$ uses a sliding window of size 110 to calculate the co-occurrence count of the given words. This count is used to compute the normalized pointwise mutual information (NPMI) between all keywords, resulting in a set of vectors for each key word. The one-set segmentation of primary queries calculate the similarity between each key query vector the aggregate of all keyword vectors. Consequently, cosine similarity is used for this similarity measurement and Coherence is the average of these similarities. The equation for NPMI is expressed as follows:

$N{PMI\left( w_{i}, w_{j} \right)}^{\gamma}=\left( \frac{PMI\left( w_{i}, w_{j} \right)}{-\log P(w_{i}, w_{j})+\epsilon} \right)^{\gamma}$	(2)

$PMI\left( w_{i}, w_{j} \right)=log\frac{P\left( w_{i}, w_{j} \right)+\epsilon}{P(w_{i})\cdot P(w_{j})}$	(3)

The probability is calculated through the consideration of the frequency of word co-occurrences, denoted as $w_{i}$ and $w_{j}$. $\epsilon$ represents a smoothing count value implemented to avoid the numerator from reaching zero and $\gamma$ determines the weight of NPMI.

…

**Evaluation metric comparison for platform-specific topic modeling models**

…

**Fig 2. Comparison of coherence values based on the number of topics from the LexisNexis data**

**Fig 3. Comparison of coherence values based on the number of topics from the Web of Science data**

”

***Newly added references***

26.	Röder M, Both A, Hinneburg A. Exploring the space of topic coherence measures. In Proceedings of the eighth ACM international conference on Web search and data mining. 2015; doi: 10.1145/2684822.2685324.

27.	Syed S, Spruit M. Full-text or abstract? examining topic coherence scores using latent dirichlet allocation. In 2017 IEEE International conference on data science and advanced analytics (DSAA). 2017; doi: 10.1109/DSAA.2017.61.

**Reviewer#3, Concern # 4:** Authors use the phrase “Topic 4 was named” etc. but do not tell how the name was given. Was it after invention of authors or was it taken from a predefined list? In the latter case, based on what criterion?

**Author response:** We appreciate your valuable comments. Based on your concern, we have added explanations and supporting references regarding topic naming. The main theme of each topic was labeled by the authors based on the top keywords with the highest importance included in the topic and from the original data. This method is predominantly used in papers utilizing topic modeling. The details are as follows.

**Author action:** We have made modifications to the parts highlighted in red in the manuscript below.

“

…

**Results of the topic analysis for LexisNexis**

In this section, the authors present the outcomes derived from applying the BERTopic model to the textual data acquired from LexisNexis. To characterize and label the identified topics, the top 8 words were extracted for each topic, as depicted in Table 3. Then the main theme of each topic was labeled by authors derived from the top keywords with the highest importance included in the topic and from the original data. This method is predominantly used in papers utilizing topic modeling algorithms [37-39].

…

**Results of the topic analysis for Web of Science**

The results obtained from employing the BERTopic on the textual data sourced from Web of Science are illustrated in this section. For each main theme, the top 8 keywords with the utmost importance were extracted to determine and assign names to the identified topics (Table 4). The naming process of main topics followed the same approach used for naming themes in LexisNexis.

”

***Newly added references***

37.	Yang Z, Wu Q, Venkatachalam K, Li Y, Xu B, Trojovský P. Topic identification and sentiment trends in Weibo and WeChat content related to intellectual property in China. Technological Forecasting and Social Change. 2022; doi: 10.1016/j.techfore.2022.121980.

38.	Boon-Itt S, Skunkan Y. Public perception of the COVID-19 pandemic on Twitter: sentiment analysis and topic modeling study. JMIR Public Health and Surveillance. 2020; 6 (4): e21978.

39.	Sharma C, Sharma S, Sakshi. Latent DIRICHLET allocation (LDA) based information modelling on BLOCKCHAIN technology: a review of trends and research patterns used in integration. Multimedia Tools and Applications. 2022; 81 (25): 36805-36831.

**Reviewer#3, Concern # 5:** The authors used unigrams to describe themes/topics/clusters. Why did they not use bi-grams, tri-grams which could be more informative and possibly better justify the names given to clusters?

**Author response:** We appreciate your valuable comments. We have considered using bigrams or trigrams. However, according to previous studies, considering bigrams or trigrams is advantageous for capturing local context since they involve combinations of words (Khoo et al., 2003). On the other hand, unigrams are the most independent units of meaning in sentences, making them more suitable for understanding the overall context. Therefore, we opted for unigrams. Additionally, Tf-idf is a concept that utilizes the inverse proportion of a word's frequency in a specific document and the proportion of documents in which the word appears, and c-tf-idf used in BERTopic extends this concept to focus on extracting class features (Ramos, 2003). Ultimately, since we are considering individual words, unigrams are more appropriate. Below, we have added references for further information.

**Author action:** In response to the reviewer's valuable comment, we have provided the feedback and are attaching the references for your consideration.

***Supporting references***

Khoo, C. S., Dai, Y., & Ee Loh, T. (2002). Using statistical and contextual information to identify two‐and three‐character words in Chinese text. Journal of the American Society for Information Science and Technology, 53(5), 365-377.

Ramos, J. (2003, December). Using tf-idf to determine word relevance in document queries. In *Proceedings of the first instructional conference on machine learning* (Vol. 242, No. 1, pp. 29-48). ISO 690.

**Reviewer#3, Concern # 6:** Figs 6 and 5 present distances between the topics. But I do not find how these distances were measured and why they fit an Euclidian 2-Dimensional plane? Or did the authors perform a linear/non-linear projection and if so, based on what criteria? I ask this as there are no scales in the figures.

**Author response:** We appreciate your valuable comments. Before addressing your valuable comments, we would like to inform you that we have reordered Figures. Now, let us provide additional explanations for these figures. Firstly, UMAP projects high-dimensional data into two dimensions by finding nearest neighbors and preserving local structure. By reducing to 2D using UMAP, the relative positions of topics can be more clearly expressed, considering the cosine distance between topic embeddings. Subsequently, the number of documents belonging to each topic is represented by the size of circles, which are placed according to the distance between topics. The size of circles indicates the relative size of each topic, reflecting its importance. We have also added this information and reference to the manuscript for better clarity. The details are as follows.

**Author action:** We have made modifications to the parts highlighted in red in the manuscript below.

“

…

**Results of the topic analysis for LexisNexis**

…

The visualization in Figs 5 and 6 confirmed both the similarity matrix and intertopic distances. Fig 5 is a heatmap that represents topic embeddings as vectors and measures the similarity between two topics by calculating the cosine similarity between each vector [26,54]. And Fig 6 is the intertopic distance plot, which utilizes UMAP to reduce topic embedding vectors to two dimensions (2D) and considers the similarity between topic embeddings using cosine distance [26,54]. In the 2D UMAP projection, the x-axis and y-axis represent the dimensionally reduced representations of the data. Subsequently, the circles are placed based on the distances between topics, and the size of each circle reflects the relative size of each topic, thus indicating the relative importance of each topic. Through those visualization, the relative importance of each topic can be easily identified and visually understood. The authors could examine the similarity between topics through the heatmap in Fig 5.양식의 맨 위

”

***Newly added references***

54.	Sievert C, Shirley K. LDAvis: A method for visualizing and interpreting topics. In Proceedings of the workshop on interactive language learning, visualization, and interfaces. 2014; 63-70.

**Reviewer#3, Concern # 7:** The similarity measurers used to create figures 4 and 7 are also unexplained. So we need a definition of distances, of similarities, and how they apply to clusters (are they measured between the cluster center and objects, as average, or 75% point or anything else).

**Author response:** We appreciate your valuable comments. We appreciate your valuable comments. Prior to addressing your valuable comments, we wish to notify you that we have rearranged the figures. Now, we would like to offer additional explanations for these figures. The heatmap illustrates the similarity between two topics by representing topic embeddings as vectors and calculating the cosine similarity between each vector pair. Because it involves cosine similarity computation between embedding vectors, the operation is angle-based. We have provided additional clarification and reference on this matter. The details are as follows.

**Author action:** We have made modifications to the parts highlighted in red in the manuscript below.

“

…

**Results of the topic analysis for LexisNexis**

…

The visualization in Figs 5 and 6 confirmed both the similarity matrix and intertopic distances. Fig 5 is a heatmap that represents topic embeddings as vectors and measures the similarity between two topics by calculating the cosine similarity between each vector [26,54]. And Fig 6 is the intertopic distance plot, which utilizes UMAP to reduce topic embedding vectors to two dimensions (2D) and considers the similarity between topic embeddings using cosine distance [26,54]. In the 2D UMAP projection, the x-axis and y-axis represent the dimensionally reduced representations of the data. Subsequently, the circles are placed based on the distances between topics, and the size of each circle reflects the relative size of each topic, thus indicating the relative importance of each topic. Through those visualization, the relative importance of each topic can be easily identified and visually understood. The authors could examine the similarity between topics through the heatmap in Fig 5.양식의 맨 위

”

***Newly added references***

54.	Sievert C, Shirley K. LDAvis: A method for visualizing and interpreting topics. In Proceedings of the workshop on interactive language learning, visualization, and interfaces. 2014; 63-70.

**Reviewer#3, Concern # 8:** I do not see any statistical analysis in the paper, but it would be interesting to know if there are statistically significant differences between within and between cluster similarities of documents. Basic statistics for data should be provided, including: cluster cardinality, mean / median similarity and standard deviation of similarity for each entire set and for the individual clusters. Same would be welcome for diversity and coherence.

**Author response:** We appreciate your valuable comments. Taking your concern into consideration, we have added statistical analysis of evaluation metrics (diversity, coherence values) and cluster cardinality. Additionally, we have provided statistical analysis of topic similarity. The details are as follows.

**Author action:** We have made modifications to the parts highlighted in red in the manuscript below.

“

…

**Evaluation metric comparison for platform-specific topic modeling models**

…

**Table 1. Statistical comparison of evaluation metrics among topic models**

| **Target platform** | **Metric**  **Method** | **Diversity** | | | **Coherence values (c_v)** | | |
| --- | --- | --- | --- | --- | --- | --- | --- |
|  |  | **Mean** | **Median** | **Standard deviation** | **Mean** | **Median** | **Standard deviation** |
| **LexisNexis** | **LDA** | 0.6772 | 0.6750 | 0.0709 | 0.3280 | 0.3032 | 0.0789 |
|  | **NMF** | 0.3684 | 0.3750 | 0.0616 | 0.2366 | 0.2377 | 0.0140 |
|  | **CTM** | 0.8944 | 0.8450 | 0.0781 | 0.3864 | 0.3928 | 0.0443 |
|  | **BERTopic** | 0.8579 | 0.8610 | 0.0902 | 0.4544 | 0.4462 | 0.0268 |
| **Web of Science** | **LDA** | 0.6891 | 0.6931 | 0.0677 | 0.3493 | 0.3644 | 0.0592 |
|  | **NMF** | 0.3914 | 0.4167 | 0.0859 | 0.2892 | 0.2934 | 0.0117 |
|  | **CTM** | 0.8961 | 0.8450 | 0.0767 | 0.3640 | 0.3673 | 0.0415 |
|  | **BERTopic** | 0.9047 | 0.8840 | 0.0329 | 0.4389 | 0.4552 | 0.0413 |

…

**Results of the topic analysis for LexisNexis**

…

**Table 3. Topic representations of LLM from LexisNexis data**

| **Topic** | **Main theme** | **Document count** | **Top keywords with the highest importance** |
| --- | --- | --- | --- |
| **0** | Possibility of using AI technology in a wide range of fields | 1,678 | (‘ai’, 0.0421) (‘chatgpt’, 0.0294) (‘technology’, 0.0216) (‘student’, 0.0196) (‘use’, 0.0188) (‘chatbot’, 0.0182) (‘work’, 0.0167) (‘human’, 0.016) |
| **1** | Spread of GPU market and LLM company | 934 | (‘ai’, 0.0444) (‘company’, 0.0346) (‘chip’, 0.0229) (‘nvidia’, 0.0224) (‘market’, 0.0217) (‘openai’, 0.0209) (‘chatgpt’, 0.0193) (‘tech’, 0.0188) |
| **2** | LLM for social media | 43 | (‘twitter’, 0.0963) (‘thread’, 0.0931) (‘user’, 0.0713) (‘musk’, 0.0541) (‘zuckerberg, 0.0453) (‘platform’, 0.0441) (‘instagram’, 0.0418) (‘linkedin’, 0.04) |
| **3** | LLM in legal cases | 41 | (‘case’, 0.0832) (‘legal’, 0.0794) (‘lawyer’, 0.0766) (‘court, 0.0702) (‘judge’, 0.0603) (‘schwartz’, 0.057) (‘cohen’, 0.0336) (‘decision’, 0.0307) |
| **4** | Youth seeking assistance from AI chatbots | 20 | (‘young’, 0.1246) (‘people, 0.0917) (‘sex’, 0.0701) (‘onlin, 0.0609) (‘nominet’, 0.0501) (‘medical’, 0.0425) (‘chatbot’, 0.0421) (‘ai’, 0.0399) |
| **5** | Altman’s World Coin launch | 18 | (‘worldcoin, 0.2125) (‘scan, 0.0994) (‘bitcoin’, 0.0793) (‘crypto’, 0.0733) (‘token’, 0.0658) (‘altman’, 0.0588) (‘cryptocurrency’, 0.0523) (‘orb’, 0.0455) |
| **6** | AI chatbot used in the Russia-Ukraine war | 17 | (‘russia’, 0.0858) (‘russian’, 0.0744) (‘putin’, 0.0534) (‘korotkov’, 0.0439) (‘fr’, 0.0439) (‘train’, 0.0399) (‘frm’, 0.0288) (‘ukraine’, 0.0279) |

…

**Results of the topic analysis for Web of Science**

…

**Table 4. Topic representations of LLM from Web of Science data**

| **Topic** | **Main theme** | **Document count** | **Top keywords with the highest importance** |
| --- | --- | --- | --- |
| **0** | Utilization of LLM across various domain | 1,146 | (‘chatgpt’, 0.0259) (‘ai’, 0.0205) (‘llm’, 0.0180) (‘learning’, 0.0174) (‘knowledge’, 0.0147) (‘processing’, 0.0145) (‘natural’, 0.0142) (‘application’, 0.0141) |
| **1** | Research on utilization of LLM in the field of education and learning | 310 | (‘student’, 0.0481) (‘school’, 0.0257) (‘english’, 0.0253) (‘teacher’, 0.0210) (‘learning’, 0.0197) (‘teaching’, 0.0195) (‘factor’, 0.0191) (‘personality’, 0.0182) |
| **2** | Research on LLM for utilizing voice recognition | 239 | (‘speech’, 0.0619) (‘effect’, 0.0309) (‘acoustic’, 0.0292) (‘speaker’, 0.0291) (‘bilingual’, 0.0256) (‘recognition’, 0.0234) (‘auditory’, 0.0211) (‘voice’, 0.0196) |
| **3** | Research on tuning LLM for improving the efficiency | 161 | (‘high’, 0.0241) (‘time’, 0.0227) (‘parameter’, 0.0197) (‘structure’, 0.0196) (‘simulation’, 0.0191) (‘flow’, 0.0187) (‘observation’, 0.0170) (‘field’, 0.0169) |
| **4** | The utilization of LLM for disease and risk detection in the medical field | 127 | (‘child’, 0.0462) (‘outcome’, 0.0361) (‘age’, 0.0329) (‘clinical’, 0.0326) (‘risk’, 0.0315) (‘group’, 0.0280) (‘treatment’, 0.0274) (‘participant’,0.0246) |
| **5** | The utilization of LLM in the field of traffic and transportation | 56 | (‘accident’, 0.0510) (‘management’, 0.0406) (‘safety’, 0.0398) (‘road’, 0.0335) (‘traffic’, 0.0278) (‘incident’, 0.0255) (‘country’, 0.0252) (‘development’, 0.0244) |
| **6** | Highlighting LLMs’ potential for assisting gesture recognition | 32 | (‘sign’, 0.2437) (‘gesture’, 0.1103) (‘hand’, 0.1015) (‘recognition’, 0.0912) (‘signer’, 0.0467) (‘image’, 0.0425) (‘slr’, 0.0344) (‘accuracy’, 0.0343) |

…

Fig 8 illustrates the distribution of distances between attained topics. There was an appropriate distance between topics without any overlap, and it was evident that Topic 0 contains a significantly larger quantity of data compared to the other topics. These visualizations suggest that, unlike news articles, the research paper derives from a single comprehensive theme and is topic-focused. Additionally, statistical analysis of the similarity between topics were performed on both platforms (Table 5).

**Table 5. Statistical analysis of the similarity between topics on LexisNexis and Web of Science data**

|  | **Similarity between topics** | | |
| --- | --- | --- | --- |
| **Platform** | **Mean** | **Median** | **Standard deviation** |
| **LexisNexis** | 0.8466 | 0.8583 | 0.0593 |
| **Web of Science** | 0.8082 | 0.8054 | 0.0652 |

”

**Reviewer#3, Concern # 9:** Have the authors made all data underlying the findings in their manuscript fully available? The authors declare that: No - some restrictions will apply.

**Author response:** We appreciate your valuable comments. We have uploaded minimal anonymized data to a public repository. Furthermore, we will detail this in the data availability section.

**Author action:** We have uploaded minimal anonymized data at public repository. Additionally, we intend to describe this in the data availability.
